# Morphological Plasticity of Emerging Purkinje Cells in Response to Exogenous VEGF

**DOI:** 10.3389/fnmol.2017.00002

**Published:** 2017-01-30

**Authors:** Leonard Herrfurth, Verena Theis, Veronika Matschke, Caroline May, Katrin Marcus, Carsten Theiss

**Affiliations:** ^1^Medizinische Fakultät, Institut für Anatomie, Abteilung für Cytologie, Ruhr-Universität BochumBochum, Germany; ^2^Abteilung für Medizinische Proteomik/Bioanalytik, Medizinisches Proteom-Center, Ruhr-University BochumBochum, Germany

**Keywords:** Purkinje cell, VEGFR-2, dendritogenesis, confocal laser scanning microscopy, microinjection

## Abstract

Vascular endothelial growth factor (VEGF) is well known as the growth factor with wide-ranging functions even in the central nervous system (CNS). Presently, most attention is given to the investigation of its role in neuronal protection, growth and maturation processes, whereby most effects are mediated through VEGF receptor 2 (VEGFR-2). The purpose of our current study is to provide new insights into the impact of VEGF on immature and mature Purkinje cells (PCs) in accordance with maturity and related receptor expression. Therefore, to expand our knowledge of VEGF effects in PCs development and associated VEGFR-2 expression, we used cultivated organotypic cerebellar slice cultures in immunohistochemical or microinjection studies, followed by confocal laser scanning microscopy (CLSM) and morphometric analysis. Additionally, we incorporated in our study the method of laser microdissection, followed by quantitative polymerase chain reaction (qPCR). For the first time we could show the age-dependent VEGF sensitivity of PCs with the largest promoting effects being on dendritic length and cell soma size in neonatal and juvenile stages. Once mature, PCs were no longer susceptible to VEGF stimulation. Analysis of VEGFR-2 expression revealed its presence in PCs throughout development, which underlined its mediating functions in neuronal cells.

## Introduction

Senger et al. (1983) first described the vascular endothelial growth factor (VEGF) as a tumor-secreted vascular permeability protein. VEGF-A, in the following referred to as VEGF, is the founding member of a homodimeric disulfide bound glycoprotein family, completed by VEGF-B, -C, -D, -E, -F and the placenta growth factor (PIGF; Neufeld et al., 1999; Ferrara et al., 2003). At least nine different isoforms of VEGF are known (VEGF_121, 145, 148, 162, 165, 165b, 183, 189, 206_), generated through alternative mRNA splicing of the VEGF gene (Robinson and Stringer, 2001; Takahashi and Shibuya, 2005; Beazley-Long et al., 2013), whereby VEGF-A_165_ is the most abundant and biologically active form in mammalian species (Ferrara et al., 2003).

VEGF molecules are able to bind to different receptors such as VEGFR-1, -2 (KDR) and -3 (*FLT-1, FLK-1, FLT-4*), which belong to the family of receptor tyrosin kinases (Neufeld et al., 1999). These transmembrane proteins contain a tyrosin kinase sequence in their intracellular part, which is interrupted by a kinase insert domain, a transmembrane region and an extracellular part with seven immunoglobulin-like domains (Ferrara et al., 2003). VEGFR-3 binds VEGF-C and VEGF-D, and is mainly expressed in lymphatic endothelium where it is responsible for inducing lymphangiogenesis (Veikkola et al., 2001). Furthermore, VEGFR-3 regulates developmental processes and adult neuronal function in the cerebellum (Hou et al., 2011). The function of VEGFR-1 is still under debate, as it binds VEGF-A, but also PIGF and VEGF-B. VEGFR-1 is likely to function as a decoy-receptor to transmit mitogenic signals (Ferrara et al., 2003), and is important in pathological conditions such as inflammation and cancer (Takahashi and Shibuya, 2005). VEGF-A_165_ and its corresponding VEGFR-2 mediate endothelial survival and growth, but are also strongly expressed in the nervous system (Takahashi and Shibuya, 2005; Zachary, 2005). The small molecule axitinib is a tyrosine kinase inhibitor, which is able to perturb VEGF pathways by specifically blocking the tyrosine residue 1214 (tyr 1214) phosphorylation of the VEGF-receptors. As a consequence, axitinib inhibits signal transduction mediated through the VEGFR-2 as well as VEGFR-1 and -3 (Kelly and Rixe, 2009).

As an angiogenic, endothelial, cell mitogen and survival factor, VEGF is best known for its role in angiogenesis and vasculogenesis (Ferrara et al., 2003; Shibuya, 2008). VEGF is subsequently expressed under conditions such as hypoxia, ischemia and injury (Shweiki et al., 1992; Banai et al., 1994). It promotes vascular development, permeability and endothelial outspreading (Senger et al., 1983; Roberts and Palade, 1995).

Currently, there is a high interest in the impact of VEGF on the nervous system. Other than its important role in neuronal proliferation and migration (Zhu et al., 2003; Ruiz de Almodovar et al., 2010), VEGF promotes neuron survival, regeneration, neurite outgrowth, growth cone guidance, axonal and neuronal growth and maturation (Sondell et al., 1999; Khaibullina et al., 2004; Ruiz de Almodovar et al., 2009; Foehring et al., 2012; Olbrich et al., 2013). Besides the neurogenic, neuroprotective and additional neurotrophic impact on the nervous system (Sondell et al., 1999; Rosenstein et al., 2003; Zachary, 2005; Beazley-Long et al., 2013), an increasing number of studies are focusing on VEGF as a stimulator of neurogenesis during development and adulthood (Jin et al., 2002; Fabel et al., 2003; Schänzer et al., 2004).

Moreover, a recent study detected a positive effect of VEGF on the dendritogenesis of interneurons in the olfactory bulb (Licht et al., 2010). Cvetanovic et al. (2011) investigated the impact of VEGF inhibitors on the survival and dendritic outgrowth of neonatal Purkinje cells (PCs) using primary cultures of postnatal day 1 (p1) spinocerebellar ataxia 1 (SCA1) mice cerebella. These experiments pointed out that low levels of VEGF negatively influenced dendritogenesis and survival of PCs. However, heretofore, few studies were published suggesting that VEGF has a particular role in neuronal plasticity, neurite maturation and dendrite formation.

To gain a deeper insight into the role of VEGF for the development of cerebellar tissue, especially on PC dendritogenesis and spinogenesis, our study was performed with organotypic cerebellar slice cultures. During PC growth, the morphology of these principle cerebellar neurons changes dramatically, which starts during embryonic development and proceeds into the juvenile stage. At birth PCs are unpolarized neurons, with small soma and multipolar perisomatic dendrites (McKay and Turner, 2005). Subsequently, polarized neurons with one or two main branches are established, which become more and more ramified during maturation. Matured PCs display a huge, unipolar, highly branched dendritic arbor covered with numerous dendritic spines. After 30 days of development qualitative but almost no quantitative changes of dendritic arbor morphology are detectable (Altman, 1972; Berry and Bradley, 1976).

It is most likely that VEGF is indeed involved in the physiological maturation process of PCs. For clinical reasons the possible impact of VEGF on adult PC has become a point of focus. It is of significant therapeutic interest whether VEGF is able to induce growth and maturation in PCs during certain developmental stages. Thus, one aim of the current study was to investigate, whether dendritogenesis, somatogenesis and spinogenesis in neonatal, juvenile and mature PCs can be stimulated by VEGF. Besides this we investigated the differential expression of VEGFR-2 in neonatal, juvenile and adult PCs by employing methods of immunohistochemistry, *in situ* hybridization and laser microdissection, followed by quantitative polymerase chain reaction (qPCR). Finally, the effects of VEGF on PCs were examined to find out whether they are inducible through molecular mechanisms of the VEGFR-2 signaling pathway by blocking studies combined with morphometric analyses.

## Materials and Methods

### Cell Cultures

Primary cerebellar slice cultures (stages p6–p30) were prepared as described previously (Meller et al., 2005; Wessel et al., 2014). All procedures were conducted under established standards of the German federal state of Northrhine Westphalia, in accordance with the European Communities Council Directive 2010/63/EU on the protection of animals used for scientific purposes.

The studies have been performed under the terms of the German animal protection law. Wistar rat pups at postnatal day 1 and day 9 (p1, p9) were used to obtain primary cerebellar slice cultures according to the roller tube technique (Wessel et al., 2014). Rat pups were decapitated, followed by dissection of the brain. Cerebella were isolated from the forebrain and brainstem, and the pia mater and superficial blood vessels were removed under a binocular microscope. Using the McIlwain tissue chopper, cerebella were cut with a razor blade into parasagittal slices with a thickness of 275 μm. After chopping, these slices were separated carefully and attached to collagen-coated (C7661; Sigma-Aldrich) glass cover slips (32 mm; Kindler, Freiburg, Germany) with 10 μl plasma (P3266; Sigma-Aldrich) and 10 μl thrombin (605157; Calbiochem). After coagulation, each cover slip was placed into a roller tube with an additional 1.3 ml nutrient medium. The medium contained 25% heat-inactivated fetal horse serum (S9135; Biochrom), 15% Hanks-Medium (H1641; Sigma-Aldrich), 6.5 mg/ml glucose, basal medium eagle (BME, B1522; Sigma-Aldrich), 0.01g/ml penicillin (P3032; Sigma-Aldrich), 0.01g/ml L-glutamine (G7513; Sigma-Aldrich), and 25 ng/ml nerve growth factor (NGF-7S, N0513; Sigma-Aldrich). Finally, these slice cultures were placed in a roller-drum incubator at a temperature of 37°C, which rotated slowly at the speed of four rounds per minute.

After 6 days *in vitro* (div) a mitosis inhibitor mixture (0.33 mM Uridine, 0.33 mM Cytosine-ß-D-arabinofuranoside hydrochloride, 0.33 mM 1-(2-Desoxy-ß-D-ribofuranosyl)-5-fluoruracil; U3003, C6645; Sigma-Aldrich; Serva 21555) was added to the slice cultures for 24 h to inhibit fibroblast proliferation, whereas after the 7th div nutrient medium was used, with a reduced heat-inactivated fetal horse serum concentration (15%). During the incubation period of up to 21 div, the nutrient medium was replaced twice per week.

### Experimental Groups

Experiments were performed with three different cerebellar slice culture groups to examine the impact of VEGF on neonatal (group 1: p1 + 5 div), juvenile (group 2: p1 + 16 div) and mature PCs (group 3: p9 + 21 div). After these specific incubation times, the method of microinjection or immunohistochemistry were performed.

To be able to use the microinjection technique, slice cultures have to flatten for a minimal amount of 2 weeks *in vitro*. To investigate developing PCs we chose the stage p1 plus 16 div PCs as the juvenile experimental group. For the analysis of mature PCs we had to use p9 rats since it is known to achieve best possible results in regard to cell survival (Krah and Meller, 1999). To analyze the effects on dendritic maturation in these cultures an additional period of 21 div is meaningful, in which the PCs are able to develop their dendritic shape.

### *In Vitro* Treatment of PCs

The impact of VEGF (V4512, Sigma Aldrich) on the morphology of PC dendrites was investigated in cerebellar slice cultures of p1 rats. In comparison to controls, slice cultures were incubated with VEGF (*c* = 0.1 μg/ml) nutrient medium as described previously (Jin et al., 2006), with a mixture of VEGF (*c* = 0.1 μg/ml) and axitinb (*c* = 0.01 mg/ml), or solely with axitinib (*c* = 0.01 mg/ml) for 48 h. Afterwards they were fixed with 4% paraformaldehyde (PFA) for 1 h and washed with phosphate-buffered saline (PBS).

### Cryosection of Rat Cerebella (p6, p17, p30)

Cryosection of rat cerebella were obtained from p6, p17 and p30 Wistar rats. After decapitation and dissection of the brain, p17 and p30 rats were fixed with 4% PFA. Cerebella were then isolated and post fixated with 4% PFA at 4°C overnight. After PBS washing, cerebella were transferred to 30% sucrose for 2 days. Afterwards, they were deep-frozen in isopentane at −50°C. Samples for cryosectioning were fixed with tissue freezing medium (Leica) and cut on a cryostat (Leica CM 3050 S; chamber and stage temperature of −18°C and −20°C respectively). Cryosections (40 μm thick) were applied on glass slides (J1800AMNZ, Menzel-Gläser, Braunschweig, Germany).

### *In Situ* Hybridization

Surgical procedures and cryosections (15 μm) of rat cerebella were obtained from p6 and p30 Wistar rats under sterile RNase-free conditions as described in Pieczora et al. (2016). All solutions were prepared with DEPC-treated water. *In situ* hybridization was performed according to the instruction manual “FFPE *in situ* hybridization using double-labeled Fluorescein or DIG miRCURY LNA^TM^ microRNA Detection probes” (Exiqon). Briefly, the cryosections were incubated in 4% PFA at room temperature for 15 min. After washing 3× 3 min with PBS the slides were incubated with 20 μg/ml proteinase K (microRNA ISH Buffer Set #9000; Exiqon) for 10 min at 37°C. For hybridization the tissue was incubated with 80 nM of double DIG-LNA^TM^ mRNA probe (VEGFR-2 custom LNA mRNA detection probe, Exiqon) probe diluted in microRNA ISH Buffer (#9000, Exiqon). The tissue was incubated for 2 h at 54°C. Afterwards the tissue was washed once with 5× SSC for 5 min, respectively twice with 1× SSC and 0.2× SSC for 5 min at hybridization temperature and finally with once with 0.2× SSC for 5 min at room temperature. To visualize the probe the slides were incubated for 1 h with anti-Digoxigenin Alkaline Phosphatase, fab fragments (sheep, 1:800, 11093274910; Roche). Meanwhile a NBT-BCIP tablet (11697471001; Roche) was dissolved in DECP-water, adding Levamisol (31742; Fluka) at the end. The AP substrate was incubated for 90 min in the dark at room temperature. Finally the tissue was incubated with 150 μl Nuclear Fast Red (N3020; Sigma-Aldrich) per slide for 1 min, washed, dehydrated and fixed with mounting medium. The tissue was stored over night at room temperature and analyzed by light microscopy (Olympus BX 61) the subsequent day.

### Immunohistochemistry

#### p1 Rat Cerebellar Slice Cultures (Group 1 + 2)

After cultivation of the cerebellar slice cultures for the appropriate time the slices were fixed in 4% PFA in PBS for 1 h and rinsed with PBS. To block non-specific binding sites and to permeabilize the cell membrane a solution of 10% goat serum (G9023; Sigma-Aldrich) and 0.2% Triton-X-100 (T8532; Sigma-Aldrich) in PBS was added for 90 min, followed by incubation with primary anti-calbindin-D28K antibodies (rabbit, 1:1000, C2724; Sigma-Aldrich) to label specifically PCs. In neonatal PCs (group 1) antibodies were diluted in 0.2% Triton-X-100 and 1% goat serum in PBS, and placed in a fridge (4°C) for 2 days. After washing with PBS, secondary anti-rabbit Alexa Fluor 488 antibodies (goat, 1:400, A11008; Molecular Probes) were used in 0.1% Triton-X-100 and 1% goat serum in PBS were applied overnight at 4°C. Juvenile PCs of group 2 were stained with anti-calbindin-D28K antibodies (mouse, 1:200, L9848; Sigma-Aldrich) diluted in PBS containing 0.1% Triton-X-100 and 1% goat serum were applied overnight at 4°C. After several washing steps with PBS, secondary anti-mouse IgG TRITC (goat, 1:200, T5393; Sigma-Aldrich) antibodies, which had been dissolved in 0.1% Triton-X-100 and 1% goat serum in PBS, were applied for 3 h. After intensive washing, nuclear counterstaining was done with bisBenzimide Hoechst 33342 trihydrochloride (1:1000, B2261; Sigma-Aldrich) for 20 min. Finally, cell cultures were rinsed with PBS and embedded in fluorescence mounting medium under a coverslip (S3023; Dako).

#### p6, p17 and p30 Cerebellar Rat Cryosections

After blocking and permeabilization, cryosections were incubated with primary antibodies against VEGFR-2 (rabbit, polyclonal, 1:100, ab39256; Abcam) and placed in a fridge (4°C) overnight. After washing with PBS, secondary FITC-coupled anti-rabbit IgG (goat, 1:400, F6005; Sigma Aldrich) or Alexa Fluor 488-coupled anti-rabbit IgG (goat, 1:400, A-11008, Molecular Probes) antibodies were added overnight at 4°C. Nuclear counterstaining was done with bisBenzimide Hoechst 33342 trihydrochloride (1:1000, B2261; Sigma-Aldrich). Further steps of immunohistochemistry were done in the same manner as just described (group 1 + 2) to label PCs.

### Microinjection (Group 2 + 3)

With aid of the microinjection technique yellow fluorescent protein-actin plasmids (pEYFP-actin; 0.195 μg/μl; custom made) were injected into single juvenile (group 2) and mature (group 3) PC cell nuclei of cerebellar slice cultures. Either sterile glass capillaries (diameter: 0.2–0.5 μm, Femtotips; Eppendorf, Germany) or capillaries (Hilgenberg, Nr. 1403512, Germany; borosilicate glass: 1.5 mm/0.2 mm; Puller: Sutter Instruments P97, USA) were filled with 2 μl of plasmid solution. Using an inverted microscope equipped with long-distance phase-contrast optics (Axiovert 35, Zeiss, Germany), PCs were imaged in slice cultures. During microinjection cultures were kept at 37°C on a heating stage. Application settings of the pressure injection devices (InjectMan NI2 and FemtoJet; Eppendorf) were set to inject with 80 hPa for 0.5s The constant pressure was defined as 60 hPa. After microinjection, nutrient medium was replaced carefully and slice cultures then further incubated for at least 24 h to receive a sufficient signal.

### Morphometric Analysis

#### Dendritic Length of Neonatal and Juvenile PCs (Group 1 + 2)

To investigate the effect of VEGF on neonatal and juvenile PCs, morphometric analysis of group 1 and 2 were performed with four different experimental conditions. All slices were cultured for 5 div (group 1) or for 16 div (group 2). The control group was incubated without any addition of VEGF or axitinib. In the other groups VEGF, VEGF + axitinib or solely axitinib were added during the last 2 days of incubation. Thereafter morphometric analysis was done with confocal laser scanning microscopy (CLSM; Axiovert 100M, LSM 510 meta; Zeiss) in combination with oil immersion lenses (Plan-Neofluar 40x/1.3 Oil, Zeiss; resolution *x*: 0.22 μm, *y*: 0.22 μm, *z*: 0.26 μm). With the aid of the Zeiss physiology kit, every single dendrite of the PCs was measured, and each distance was added together to give the total dendritic length. To determine morphometric changes measured data of the control group were defined as 100% whereby the changes were calculated. Statistical analysis of the impact of VEGF on neonatal and juvenile PC morphology was performed with 320 PCs from a pool of at least 25 rat cerebella.

#### Dendritic Spreading, Dendritic Length and Cell Soma Area of Juvenile and Mature PCs

To examine the impact of VEGF on the dendritogenesis of juvenile and mature PCs, morphometric analysis of group 2 and 3 were performed after exposure to different experimental conditions. After microinjection of pEYFP-actin into PCs of group 2 and group 3, CLSM was done (Plan-Neofluar 20x/0.5; Zeiss; resolution *x*: 0.22 μm, *y*: 0.22 μm, *z*: 1.3 μm). Control slices were cultured without the addition of VEGF or axitinib. The other slice cultures were cultured with VEGF (group 2 + 3), VEGF + axitinib (group 2) or solely axitinib (group 2) for 48 h. Thereafter the same PCs were studied (*n* = 20 per condition). Using the Zeiss physiology kit, dendritic length was measured as described before. Circumference of the dendritic arbor and cell soma area were measured by manual encircling, with the measured changes quoted as percentages. Statistical analysis of the impact of VEGF on juvenile and mature PC morphology was performed with 120 PCs from a pool of at least 80 rat cerebella.

### Quantitative PCR (qPCR)

#### Surgical Procedures/Cryosection for Laser Microdissection

Surgical procedures and cryosections of rat cerebella were obtained from p6 and p30 Wistar rats under sterile RNase-free conditions as described in Pieczora et al. (2016). After decapitation and dissection of the brain, cerebella were isolated and frozen at −50°C in isopentane and stored at −80°C. For affixing the tissue to the cryostat (Leica CM 3050 S), a minimal amount of freezing medium (Leica) was used. Samples were cut at a thickness of 10 μm, placed on glass slides coated with polyethylene napthalate (PEN; 11505189; Leica) and stored at −80°C.

#### Cresyl Violet Staining

After being left to thaw at room temperature, the slides were placed in 70% p.a. ethanol for 2 min followed by incubation in 1% cresyl violet solution in 50% p.a ethanol for 30 s. Sections were then dehydrated first in 70% p.a. ethanol several times and then in 100% p.a. ethanol for 10 s. Finally, the slides were dried at room temperature. All solutions were diluted in DEPC-treated water.

#### Laser Microdissection

Laser microdissection was performed with a PALM Micro Beam instrument (P.A.L.M.-System LCM, Carl Zeiss Microscopy GmbH) with non-adhesive tubes (MicroTube 500, Carl Zeiss Microscopy GmbH) as described previously by Molina et al. (2015). In brief, laser microdissection was done with 10 μm-thick cresyl violet stained cryosections. The optimal thickness was based on the ability to catapult the regions of interest after laser-cutting. These regions were visualized in the tissue sections and marked for the laser beam within the software provided with the instrument. The non-adhesive tube (filled with 50 μl of lysis buffer, Z6010, Promega) was placed over the section and laser microdissection was performed. In total about 3000 PCs were collected. Afterwards, the tube was closed and stored upside-down at −80°C until further usage.

#### RNA Isolation and qPCR

Total RNA was extracted from PC-enriched samples (around 3000 laser microdissected cells), initially filled up to 300 μl lysis buffer (Z6010, Promega), using ReliaPrep^TM^ RNA Cell Miniprep System (Z6010, Promega). Briefly, cells were homogenized in 300 μl lysis buffer with 2% 1-Thioglycerol added. Nucleic acids were bound to ReliaPrep^TM^ Minicolumns by centrifugation. For removal of genomic DNA an on-column DNAse digestion was performed according to manufacturer’s description. Total RNA was eluted with 15 μl nuclease free water and frozen at −80°C for storage. cDNA synthesis was performed using Reverse Transcription System (A3500, Promega) according to manufacturer’s protocol using 1 μg total RNA and Oligo(dT)15 Primers. Reaction mixture was incubated for 60 min at 42°C and stopped by heating the sample at 95°C for 5 min following an incubation at 4°C for 5 min. cDNA was stored at −20°C until use. Standard quantitative PCRs were performed on a CFX96 Real Time PCR Detection System (BioRad) using GoTag^®^ qPCR Master Mix (A6001, Promega) according to manufacturer’s recommendation. Melting curves were obtained after each PCR run and showed single PCR products. Expression levels for the genes of interest and for housekeeping gene *GAPDH* were measured in three independent PCR runs. Fold change of expression was calculated using the 2^−ΔΔCt^ method. The relative expression levels of validated mRNAs were compared using an unpaired two-tailed *t*-test. In order to visualize the purity of microdissected PCs, the relative mRNA amount of the marker genes was expressed as a percentage. We added the relative means of all marker genes and defined the amount as 100%. We were then able to generate the relative amount by calculating the ratio of the marker genes compared to the total relative amount of mRNA (Pieczora et al., 2016). Primer sequences used were: *KDR* (codes for VEGFR-2): (5′-TCC CAG AGT GGT TGG AAA TG-3′, 3′-ACT GAC AGA GGC GAT GAA TG-5′, Microsynth); *FLT1* (codes for VEGFR-1): (5′-GTG AAG AGT GGG TCG TCA TTC-3′, 3′-CTA TGG TTT CCT GCA CCT GTT-5′); *GAPDH*: (5′-ACT CCC ATT CTT CCA CCT TTG-3′, 3′-CCC TGT TGC TGT AGC CAT ATT-5′, Microsynth). To analyze distinct mRNAs in microdissected PCs we compared different neuronal marker genes. The following primers were used: *calbindin* (5′-GAA GGA AAG GAG CTG CAG AA-3′, 5′-TCT GCC CAT ATT GAT CCA CAA A-3′, Microsynth), *GFAP* (5′-GAG TGG TAT CGG TCC AAG TTT-3′, 5′-TTG GCG GCG ATA GTC ATT AG-3′, Microsynth), *NeuN* (5′-GAG AAG CTC AAT GGG ACG AT-3′, 5′-CAT ATG GGT TCC CAG GCT-3′, Microsynth), *TNFα* (5′-GCT CCC TCT CAT CAG TTC CA-3′, 5′-GCT ACG GGC TTG TCA CTC-3′, Microsynth), *α6GABAa-R* (5′-CCG ATG AGA CTG GTT AAC TTC C-3′, 5′-TCT TCT GGG ACC TCT ACT GAA T-3′, Microsynth), *VGlut1* (5′-CCT GCG CAG TCG TCA TAT AAT-3′, 5′-GCC CTT GGA GTG TGA GTA TC-3′, Microsynth), and *GAPDH* (5′-ACT CCC ATT CTT CCA CCT TTG-3′, 5′-CCC TGT TGC TGT AGC CAT ATT-3′, Microsynth). For statistical analysis, Microsoft Excel and Graph Pad Prism were used.

## Results

### Morphometric Analysis of PCs After VEGF Treatment

**Figure 1:**
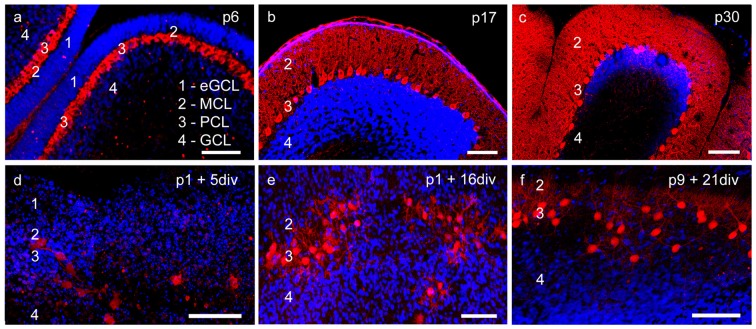
**Cerebellar cortex structure in cryosections and organotypic slice cultures. (A–C)** Cryosections: p6, p17, p30; **(D–F)** cerebellar slice cultures: p1 + 5 div, p1 + 16 div, p9 + 21 div. **(A)** In p6 cryosection and **(D)** p1 + 5 div cerebellar cultures calbindin-positive Purkinje cells (PCs; red) and cell nuclei (blue) display the four-layered cortex structure of the cerebellum. Extragranule cells (eGC) form the outermost cell layer called the external granule cell layer (eGCL). In the juvenile stage at p17, eGC migration has finished and the typical three-layered structure of the cerebellar cortex can be seen in cryosections **(B)** and cerebellar slice cultures **(E)**. **(C,F)** In mature PCs, dendritic trees extend into the thick molecular cell layer (MCL), while the Purkinje cell layer (PCL) is characterized by the line-shaped arrangement of PC somata. Also, in the organotypic slice cultures, the three-layered structure of cerebellar cortex is still well retrained. Scale bars: 100 μm.

**Figure 2:**
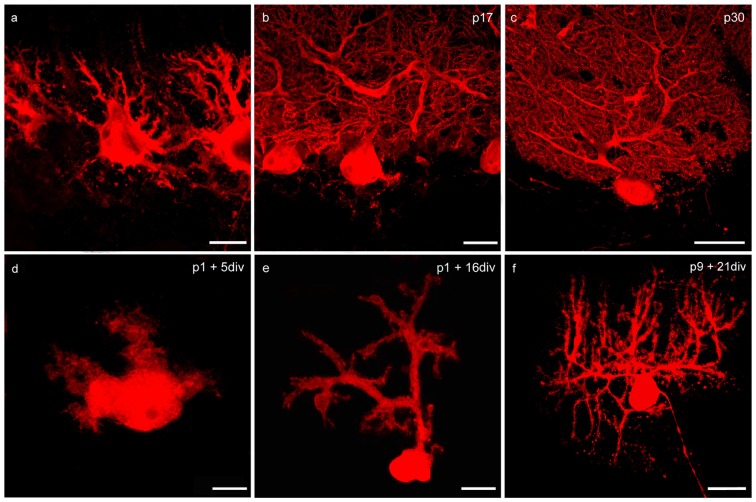
**Changes of PC morphology in cryosections and slice cultures during development. (A–C)** Cryosections: p6, p17, p30; **(D–F)** cerebellar slice cultures: p1 + 5 div, p1 + 16 div, p9 + 21 div. **(A)** In early development calbindin-positive PCs labeled with red fluorescent secondary antibody display a bipolar shape with perisomatic pre-dendrites. **(B)** During maturation, PCs show main and secondary dendrites until single tertiary branches can be detected at p30 **(C)**. These matured PCs are characterized by highly ramified dendritic arbors that are covered by numerous dendritic spines. In addition, in organotypic cerebellar slice cultures the development of PCs is very similar, as the dendritic arbor expands during development **(D–F)**. Scale bars: **(A,D)** 10 μm **(B,E)** 20 μm **(C,F)** 30 μm.

In our study we examined the effects of VEGF on neonatal, juvenile and mature PCs in cerebellar roller tube slices. The functionality of PCs in slice cultures was proven in former studies by analysis of axonal transport and functional synapses (Krah and Meller, 1999; Wessel et al., 2014). With aid of immunohistochemistry and CLSM our first aim was to analyze cerebellar roller tube slices in regard to their organotypic structure. Therefore, various incubation times of cerebellar slices of p1 and p9 pups were compared with cerebella cryosections of p6, p17 and p30 rats (Figures 1A–F, 2A–F).

At birth cerebellar structures are still immature, with a characteristic external granule cell layer (eGCL) above the molecular cell layer (MCL). In cryosections of p6 rat cerebella, and in cerebellar slice cultures of p1 rats cultured for 5 days, the fourth layer (eGCL) still exists (Figures 1A,D). At this early stage PCs are distinguished by multiple short perisomatic dendrites (Figures 2A,D). At this point of time granule cells (GCs) migrate from the eGCL through the MCL and Purkinje cell layer (PCL) to generate the inner granule cell layer (GCL), with its migration peak at p10-11 (Altman, 1972; Kunimoto and Suzuki, 1997), and its end after 16 days of maturation (Mancini et al., 2009). Thus, cryosections of p17 rat cerebella show the three-layered cerebellar cortex with a band of PCs, which is quite similar to the slice cultures of p1 rats incubated for 16 div (Figures 1B,E). At these juvenile stages, PCs contain one or two stem dendrites covered with a moderate number of dendritic spines (Figures 2B,E).

Matured cerebellar slices of p9 rats followed by 21 div also displayed the typical three-layered cerebellar cortex structure, which is comparable to the morphology of cryosections of p30 rats, characterized by a thick MCL with prominent dendritic arbors of the PCs. Cell bodies of PCs are arranged, as a clear band in the PCL, and densely packed GCs are located in the innermost GCL (Figures 1C,F). At this mature stage, the impressive dendritic arbor arises from the stem dendrites ramified with two or three main, and multiple smaller dendritic branches, with numerous dendritic spines attached to them (Figures 2C,F). Thus, the roller tube slices represent a functional and compact organotypic cerebellar cortex, which can be cultivated for several weeks. It is highly suitable for obtaining a deeper insight into the mechanism of VEGF-effects on PCs of the cerebellum when concerning their maturation.

### VEGFR-2 Expression in the Cerebellum Focusing on Neonatal, Juvenile and Mature PCs

To determine any impact of VEGF on PC morphology, which is likely to be mediated through an interaction with VEGFR-2, expression of this receptor was verified in neonatal, juvenile and mature PCs (p6, p17, p30) in the cerebellum. Therefore, the methods of immunohistochemistry, and additionally laser microdissection with qPCR were used.

**Figure 3:**
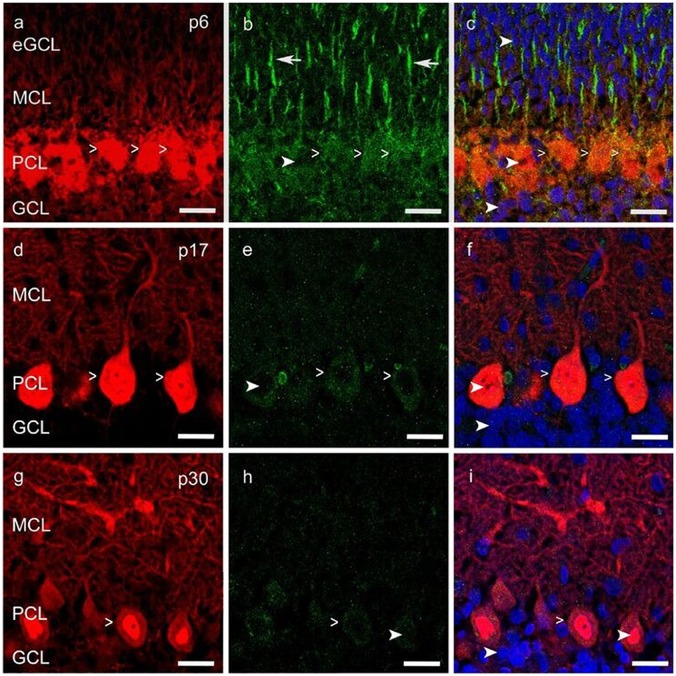
**Vascular endothelial growth factor receptor 2 (VEGFR-2) expression in cerebellar p6, p17 and p30 rats. (A-I)** Immunostaining of calbindin positive PCs (red), VEGFR-2 (green) and cell nuclei (blue) in cryosections of rat cerebella at p6, p17, p30. VEGFR-2 receptors are localized within the soma (simple white arrowheads; **A–I**) of PCs and in Bergmann glia fibers (white arrows; **B**), reaching up to the MCL; exemplarily few cell nuclei are labeled in different cell layers (curved white arrowheads; **B,C,E,F,H,I**). Scale bars: **(A–I)** 20 μm.

An intense signal of the VEGFR-2 antibody was detectable in neonatal PC cell bodies and dendrites as well as in Bergmann glia fibers, along with a diffuse signal in the eGCL, MCL and inner GCL (Figures 3A–C). In the PCL and MCL of juvenile p17 rat cerebella, positive VEGFR-2 labeling degraded in PC dendrites and cell somata (Figures 3D–F). In p30 rat cerebella with almost mature PCs, positive VEGFR-2 signals were hardly observed in the PCL (Figures 3G–I).

**Figure 4:**
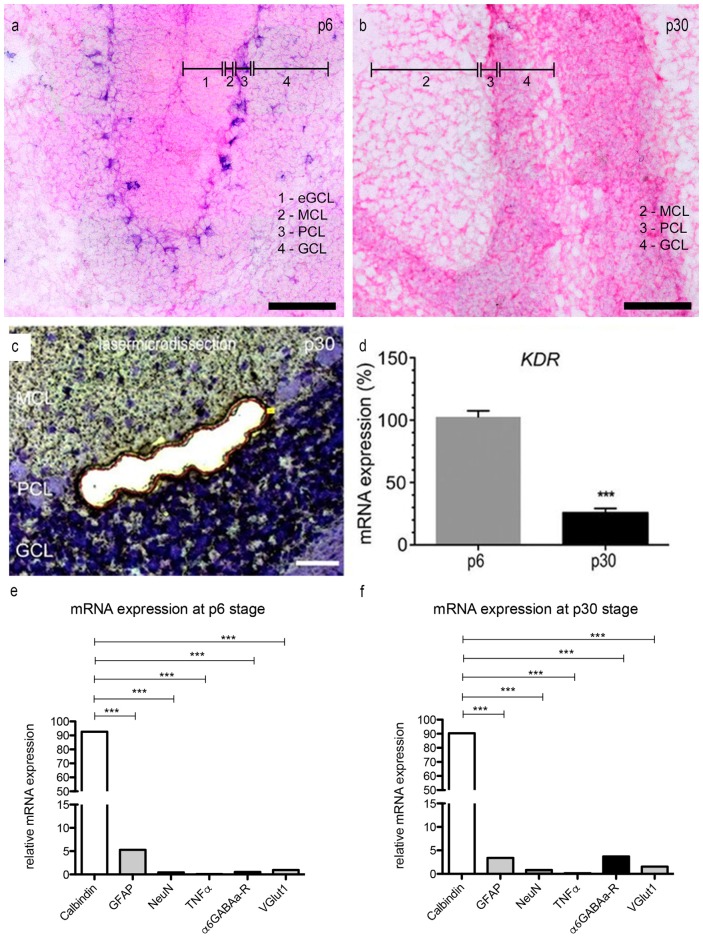
**mRNA expression levels in PCs. (A,B)** *In situ* hybridization: expression of VEGFR-2 in rat cerebella at the age of p6 **(A)** and p30 **(B)**. Cryosections (15 μm) of each stage were incubated with 80 nM double-DIG-LNA^TM^ probe (Exiqon). In p6 nearly every PC showed a positive VEGFR-2 signal. During development the expression of VEGFR-2 extremely decreases. In p30 VEGFR-2 positive PCs were hardly detectable. P6 and **(C)** p30 PCs were laser microdissected at 200× magnification and subjected to the method of quantitative polymerase chain reaction (qPCR) **(D)**. For relative quantification of *KDR* expression, the 2^−ΔΔCt^ method was conducted using the housekeeping gene *GAPDH* for normalization; data are provided as means ± SEM. Data were tested for significance using Student’s *t*-test. Significant differences are indicated by ***p < 0.0001; *n* = 3. **(E,F)** Expression of specific markers in microdissected PCs in rat cerebella at the age of p6 **(E)** and p30 **(F)** using qPCR, normalized to GAPDH. Relative expression of mRNA markers was compared by using an unpaired two-tailed *t*-test. These values were summed up and defined as 100%. The ratio of each marker mRNA was compared to the total relative expression of mRNA. This reveals a slight contamination in microdissected PCs with surrounding cells. (***p < 0.0001). Scale bars: **(A,B)** 100 μm **(C)** 50 μm.

In order to examine the expression of VEGFR-2 mRNA in PCs at the age p6 and p30 we performed *in situ* hybridization using a specific VEGFR-2 double DIG-LNA^TM^ mRNA probe (Exiqon). We detected a high expression of VEGFR-2 mRNA in very young PCs (Figure 4A). The PCL could be visualized very distinct by labeling the cell bodies and dendrites of neonatal PCs, located between the MCL and inner GCL. In p30 rat cerebella positive VEGFR-2 labeling was hardly detectable, (Figure 4B). Additionally we could reveal a positive signal in the eGCL and inner GCL in p6 rat cerebella as well as in the GCL at the stage p30. These findings demonstrate that VEGFR-2 expression extremely decreases during PC development and correspond with the results presented by immunohistochemistry.

Via the qPCR method, mRNA expression of *KDR*, that codes for VEGFR-2, was detected in microdissected PC-enriched samples of very young and mature stages (Figure 4C). Gene expression values for p6 were set to 100%. At p30 the expression level of *KDR* mRNA was decreased to only 28% (Figure 4D). Additionally we analyzed the expression of the VEGFR-1 encoding *FLT1* gene in p6 and p30 rat cerebella, setting *KDR* gene expression values for p6–100%. In p6 and p30 the expression of *FLT1* mRNA did not significantly change in young and mature PCs, the ratio amounted less than 5% (Supplementary Figure 1). Thus, laser microdissection followed by qPCR, as well as immunohistochemistry, underline that VEGFR-2 is expressed in PCs during their entire development, albeit with a significant decrease in mature stages.

To ensure that the VEGFR-2 expression is highly unique for PCs, we performed a qPCR with specific marker genes for PCs, Bergmann glia, microglia and GCs. The amount of contamination from cells other than PCs is extreme low (Figures 4E,F). At the age of p6, calbindin (a specific marker for PCs) amounts to 93.6 ± 1.2% and 90.3 ± 1.1% in p30 rat cerebella. Nevertheless, a slight contamination with neuronal and glial marker can be observed. In rat cerebella at stage p6 the relative amount of GFAP expressed mRNA is 5.3 ± 0.08%, 3.41 ± 0.05% in p30. Furthermore the contamination of gene markers for NeuN is 0.46 ± 0.01% in young rat cerebella and 0.83 ± 0.01% in p30. For TNFα the contamination is in the range of 0.1 ± 0.01% in p6 and 0.14 ± 0.01% in p30, for α6GABAa-R it is 0.54 ± 0.01% and VGlutI is 1 ± 0.01% at the age of p6. At the age of p30 the contamination for α6GABAa-R is 3.7 ± 0.02% and VGlut1 0.02 ± 0.01%.

### Impacts of VEGF on Dendritic Length, Circumference of the Dendritic Arbor and Cell Soma Area of PCs

#### VEGF Increases Dendritic Length of Neonatal PCs

**Figure 5:**
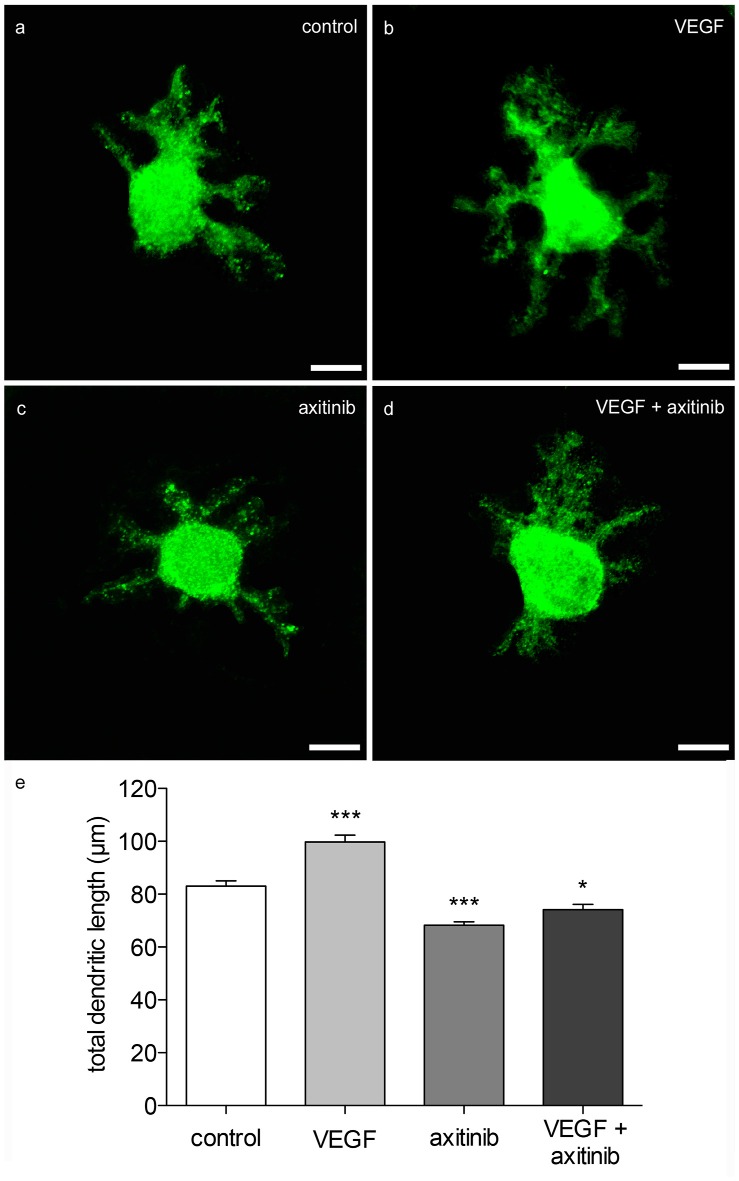
**Morphometric analysis of VEGF effects on neonatal PC dendrites. (A)** Analysis of young, calbindin-stained PCs (labeled with green fluorescent secondary antibody, p1 + 5 div), and treated with VEGF **(B)**, axitinib **(C)** or VEGF + axtinib **(D)** for a period of 48 h. **(E)** Morphometric analysis of the dendritic length. Data are provided as means ± SEM. Data were tested for significance using one-way ANOVA with Bonferroni multiple comparisons post-test. Significant differences are indicated by ***p < 0.0001, *p < 0.05; *n* = 40. Scale bars: 10 μm.

In order to visualize the effect of VEGF on neonatal PCs (group 1), cerebellar slice cultures were incubated with VEGF after 3 div for further 48 h. Besides VEGF treatment, VEGF + axitinib, or solely axitinib, were added. As controls, slices without any additive component were cultured for 5 div. Subsequently these cultures were subjected to immunohistochemistry. The length of all dendrites of one calbindin-positive PC was summed in at least 40 PCs per condition as described in Wessel et al. (2014). Here, in comparison to PC dendrites of control slices (total dendritic length averages around 83 ± 2 μm), VEGF exposure induced a significant increase in the total dendritic length of averaging 100 ± 3 μm (*p* < 0.0001; Figures 5A,B). Incubation with axitinib, which inhibits signaling pathways of VEGF-receptors such as the VEGFR-2, led to a significant reduction in the total dendritic length to 68 ± 1 μm (*p* < 0.0001) compared to the control (Figure 5C). After incubation with a mixture of VEGF plus axitinib, a significant reduction in the total dendritic length to 74 ± 2 μm was perceived in comparison to the control (*p* < 0.05; Figures 5D,E).

Thus, neonatal PCs are susceptive and sensitive to VEGF in general. It is known that physiological levels of VEGF are necessary for an undisturbed dendritogenesis process in the cerebellum, whereas experimental VEGF addition is capable of promoting and enhancing dendritogenesis of neonatal PCs. As VEGF effects were reduced by axitinib in neonatal PCs, here it is likely that the impact is mediated through signal cascades of VEGF-receptors. However, as the combination of VEGF + axitinib leads to a slightly obvious increase, dendritogenesis may also be driven by non-axitinib-sensitive pathways.

#### VEGF Increases Dendritic Length, Dendritic Tree Spreading and Cell Soma of Juvenile PCs

**Figure 6:**
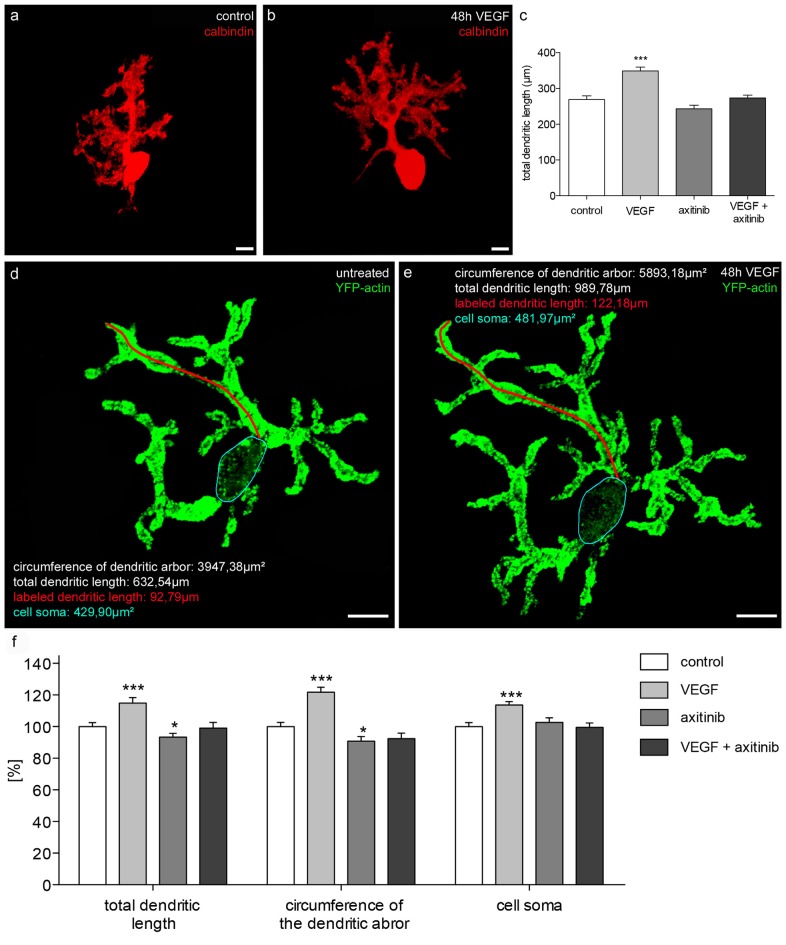
**Morphometric analysis of VEGF effects on juvenile PC dendrites and cell somata. (A)** Analysis of the total dendritic length of calbindin-stained PCs (red; p1 + 16 div), or PCs incubated with VEGF **(B)**, VEGF + axitinib or axitinib for a period of 48 h. **(C)** Morphometric analysis of the dendritic length. Data are provided as means ± SEM. Data were tested for significance using one-way ANOVA with Bonferroni multiple comparisons post-test. Significant differences are indicated by ***p < 0.0001; *n* = 40. Scale bars: 10 μm. Besides morphometric analysis of the total dendritic length (one dendrite is exemplarily labeled in red), circumference of the dendritic arbor and cell soma area (blue encircled) of pEYFP-actin microinjected PCs (green) before **(D)** and after 48 h drug incubation **(E)** are analyzed. **(F)** Changes in morphometric parameters for controls and treated cells are given in percent; data are provided as means ± SEM; *n* = 20. Untreated neurons were defined as control (100%) and treated cells were normalized to this control. Scale bars: 20 μm. Data were tested for significance using one-way ANOVA with Bonferroni multiple comparisons post-test. Significant differences are indicated by ***p < 0.0001, *p < 0.05; *n* = 20. Scale bars: 20 μm.

To investigate the effects of VEGF on juvenile PCs (group 2), cerebellar slice cultures were cultured for 16 div as controls, or for 14 days and then subjected to VEGF, VEGF + axitinib or axitinib treatment for another 2 days. The total length of immunostained PC dendrites were measured and summed to get the total dendritic length (*n* = 40 PCs per condition). A significant increase in the dendritic length to 349 ± 11 μm (*p* < 0.0001) was measured under VEGF treatment conditions compared to the control (269 ± 10 μm; Figures 6A,B). Incubation with solely axitinib or VEGF + axitinib did not lead to any significant changes of the dendritic length (Figure 6C). However, after axitinib, or VEGF plus axitinib incubation, PCs showed a significant total dendritic length reduction to 243 ± 10 μm or 274 ± 8 μm in comparison to VEGF treated PCs (*p* < 0.0001). Thus, VEGF also has the capability to enhance dendritogenesis at the juvenile stage. Besides this, single PCs of group 2 were microinjected with pEYFP-actin (Figures 6D,E). After the microinjection, individual PCs could be analyzed by CLSM before they were treated with VEGF, VEGF + axitinib or solely axitinib for another 2 days. As only individual PCs are labeled with the aid of this microinjection technique, we could identify and capture the same PC after treatment to analyze different parameters as the total dendritic length, the dendritic tree spreading and the cell soma area again (*n* = 20 PCs per condition). The huge advantage of this method is that identical cells are examined before and 2 days after experimental treatment. After 48 h of incubation untreated cells served as controls and were equated with 100%. VEGF treatment induced significant increases in the total dendritic length (15 ± 4%, *p* < 0.001), in the circumference of the dendritic arbor (22 ± 3%, *p* < 0.001) and in the cell soma area of 14 ± 2% (*p* < 0.001; Figures 6D–F). Compared to control, axitinib incubation resulted in a significant reduction in total dendritic length (−7 ± 2%, *p* < 0.05), and in circumference of the dendritic arbor (−9 ± 3%, *p* < 0.05), whereas changes in cell soma area were not significant. Growth parameters of VEGF plus axitinib-treated PCs showed a slight but not significant decrease in all parameters (Figure 6F).

Thus, with the aid of pEYFP-actin staining, our study underlines the capability of VEGF to enhance dendritogenesis in juvenile stages, probably through interaction with VEGFR-2. Remarkably, VEGF exposure induced an increase of cell soma area, although physiological cell soma growth has nearly finished at this point of time.

#### VEGF has no Impact on Dendritogenesis in Mature PCs

**Figure 7:**
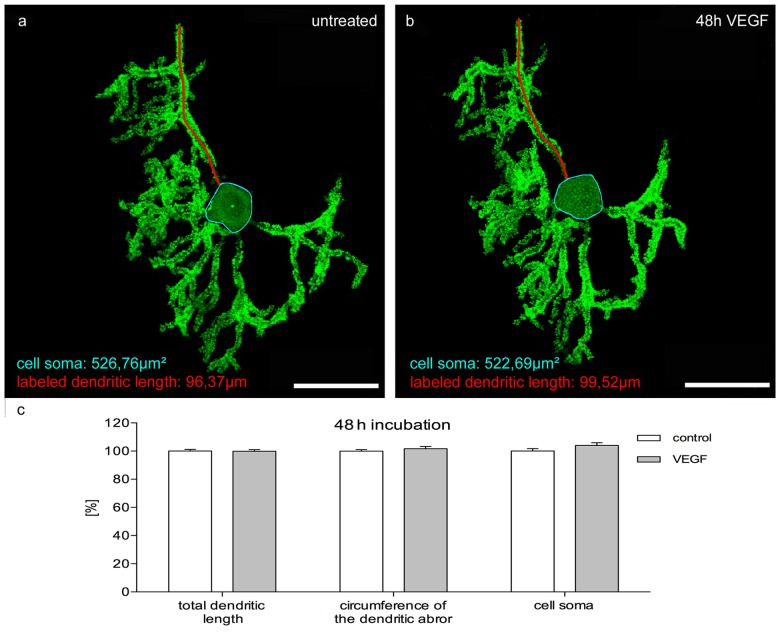
**Morphometric analysis of VEGF effects on mature PC dendrites and cell somata.** Analysis of the total dendritic length (one dendrite is exemplarily labeled in red), circumference of the dendritic arbor and cell soma area (blue encircled) of pEYFP-actin microinjected PCs after a 48 h incubation time. **(A)** Untreated PC (p9 + 21 div) and the same PC after VEGF administration for 48 h **(B)**. **(C)** Changes of morphometric parameters for controls and treated cells are given in percent; data are provided as means ± SEM. Untreated neurons were defined as control and treated cells were normalized to this control. Data compared to control were tested for significance using Student’s *t*-test. *n* = 20; Scale bars: 50 μm.

To investigate the effects of VEGF on matured PCs (group 3) in cerebellar slice cultures, pictures of native pEYFP-actin microinjected PCs were captured with the aid of CLSM. Subsequently, these cultures were incubated with or without additional VEGF for 2 days. The same PCs were imaged again and analyzed in regard to their length growth parameters as described above. In the control group, no significant changes were measured either in the total dendritic length, circumference of the dendritic arbor or in the cell soma area after 2 days of incubation. Additional VEGF exposure did not promote any significant increase in dendritic length, circumference of the dendritic arbor or cell soma area (Figure 7). Thus, at this point in time the maturation of the PC dendritic tree and cell soma has finished, and VEGF is not capable to induce dendritogenesis and somatogenesis in mature PCs.

## Discussion

After the initial description of VEGF as a tumor-secreted vascular permeability factor (Senger et al., 1983), the understanding of VEGF functions in mammalian species expanded rapidly from their central role in angiogenesis and vasculogenesis, to their acceptance as a neurotrophic factor. Despite the increasing scientific and therapeutic interest in VEGF, information on its effects in the central nervous system (CNS) is still limited. The influences of VEGF on neurons of the cerebellum were already the subject of different studies such as in granule cell migration (Ruiz de Almodovar et al., 2010). However, only sparse and incomplete information are available about the impact of VEGF on PC development and maturation. Furthermore, the mechanisms of VEGF receptor signaling within PCs are still incomplete. Thus, in the present study, effects of VEGF on neonatal, juvenile and mature PCs were analyzed in organotypic cerebellar roller-tube slice cultures, and additionally VEGFR-2 distribution on PCs was verified. Consequently, VEGF effects on PC dendritogenesis and somatogenesis were determined to be mediated through molecular mechanisms of VEGFR-2 signaling pathways.

### Neonatal, Juvenile and Mature PCs Express VEGFR-2

The impact of VEGF in the nervous system is more and more becoming an investigative focus, so that VEGF-receptor expression in the peripheral nervous system (PNS) and CNS has been investigated in several studies. Many VEGF effects are mediated through VEGFR-2 (Zachary, 2005; Carmeliet and Ruiz de Almodovar, 2013) and this receptor expression was detected in neuronal tissues of the forebrain, in the hippocampus (Yang et al., 2003; Fournier et al., 2012), but also in the subventricular zone and olfactory bulb (Maurer et al., 2003), and in the temporal cortex (Boer et al., 2008). Licht et al. (2011) assumed the possibility of low levels of VEGFR-2 expression in non-endothelial cells, confirmed by the verification of VEGFR-2 expression in for example glia cells as astrocytes or Bergmann glia (Ruiz de Almodovar et al., 2010; Wuestefeld et al., 2012). Here, low levels of VEGFR-2 expression were detected in adulthood (Kremer et al., 1997; Matsuzaki et al., 2001; Cui et al., 2010). In line with this, a decrease of VEGF and VEGFR-2 levels in the cerebellum was underlined in a former study, which demonstrated that perikarya of PCs had a positive VEGFR-2 signal during gestation, but 13 months postnatal no VEGFR-2 signal was detectable anymore (Sentilhes et al., 2010). Another study showed VEGFR-2 expression in PCs of very young primary cerebellar slice cultures of mice by immunohistochemistry, and additionally they also detected VEGFR-2 expression in cryosections of mice cerebella with aid of quantitative RT-PCR (Cvetanovic et al., 2011).

In the present study, these results could be confirmed with the detection of VEGFR-2 expression in rat PCs by immunohistochemistry and *in situ* hybridization. Here a strong positive receptor signal was detected in the young developmental stages, with a significant reduction of VEGFR-2 protein. Using laser microdissection, followed by qPCR we demonstrated the reduced expression level of VEGFR-2 mRNA in mature stages and an unchangeable low expression level of VEGFR-1. Based on the hardly detectable VEGFR-1 amounts it might be assumed that the impact of VEGF on young cerebellar PCs is most likely mediated by VEGFR-2; no visible effects on mature PCs would go along with the results of IHC and qPCR. To ensure the observed effects ascribe to age-dependent expression levels of VEGFR-2 in PCs, the mRNA level of neuronal and glial markers were analyzed in microdissected PCs. The cerebellar cortical tissue is organized very densely. PCs are surrounded by several cell types such as Bergmann glia (Verkhratsky and Reichenbach, 2010). A slight contamination of microdissected PCs with these cells is inevitable. We were able to detect calbindin, a specific marker for PCs, as the vast majority component in our microdissected samples (Baimbridge et al., 1992). Furthermore, we could exclude contamination with immunoactive microglial cells as the mRNA expression level of TNFα was hardly detectable, indicated the absence of microglial cells (Deng et al., 2008; Kaur et al., 2014). Besides this, contamination with other cerebral cell types could be excluded. The expression level of NeuN, a marker for neuronal cells of the CNS (Mullen et al., 1992), was extreme low as well as the expression levels of α6GABAa-R and VGlut1 mRNA. These findings underlined former studies which disproved the presence of α6GABAa-R and VGlut1 within the PCL in rodents. (Fremeau et al., 2001; Wojcik et al., 2004; Hörtnagl et al., 2013). The samples of microdissected PCs showed a very low amount of contamination with cells surrounding PCs. Additionally, these results reveal another important fact: the extremely low levels of GFAP and NeuN mRNA in p6 and p30 PCs, indicating a negligible impact of VEGFR-3. VEGFR-3 is upregulated in glial cells and strongly associated with the expression of GFAP and NeuN (Shin et al., 2008; Ward and Cunningham, 2015). Recently it could be shown that GFAP was highly expressed at the age of p15 in rats, accompanied with high expression of VEGFR-3. In the neonatal period the expression of NeuN and VEGFR-3 showed a comparable expression pattern (Ward and Cunningham, 2015). In our study the expression of glial and neuronal markers in microdissected PCs could be disproved in both examined stages. The purity of the microdissected samples as well as the closely connected expression of glial and neuronal markers with VEGFR-3 expression allows us to discuss the higher levels of VEGFR-2 in p6 compared to p30 in a developmental context (Brown et al., 2013; Sugino et al., 2015; Ward and Cunningham, 2015).

### VEGF Influences Neonatal and Juvenile PC Development in Cerebellar Slice Cultures—No Effects are Detectable in Mature PCs

Two to three days before birth, after PC-migration has stopped, PCs exhibit a bipolar fusiform shape that changes into a stellate shape with perisomatic predendrites. After a short retraction phase, main branches are established and the definitive polarity of these neurons is established (Armengol and Sotelo, 1991; Sotelo and Dusart, 2009). Our slice cultures of group 1 and group 2 were examined as representatives of an early developmental stage. Through the method of microinjection and immunohistochemistry it was possible to label PCs in organotypic cerebellar slice cultures and measure the total dendritic length, circumference of the dendritic arbor and soma size in different developmental stages. As it is impossible to microinject PCs in the cerebellar slices at the neonatal stage, here, solely the method of immunohistochemistry was used to investigate dendritogenesis. The present results demonstrate for the first time a significant increase in the total dendritic length of very young PCs after VEGF addition compared to control slice cultures.

Remarkably, in the prolonged maturation process of dendritogenesis and somatogenesis in PCs, VEGF also seems to be a growth factor for dendrites and cell somata. At this point in time, direct quantification of VEGF-induced changes or changes due to VEGF receptor inhibition on PC morphology was performed through the precise method of microinjection and immunohistochemical analysis. Significant additive effects in all growth parameters were measured under VEGF exposure, so that PC dendritogenesis is most likely enhanced in neonatal and juvenile stages. Therefore, together with NT3, BNDF, NGF, BMP7, Reelin or T_3_ VEGF is another highly potential stimulating and enhancing factor of dendritogenesis (Miller and Kaplan, 2003; Boukhtouche et al., 2010; Castagna et al., 2014). The effects of direct exogenous VEGF application on developing PC dendritogenesis were not yet analyzed. However, Cvetanovic et al. (2011) examined whether replenishing VEGF improves the SCA1 phenotype in mice by using clinically affected animals, which overexpressed human VEGF. Thereby, an enhancement of Rotarod performance was measured and a generally better outcome of SCA1 mice was detected, which underlined promoting and protective effects of VEGF on PCs.

Earlier studies revealed that after the initial 30 days development period, PCs reached their mature dendritic shape with tertiary dendritic branches, with no further quantitative changes detected (Altman, 1972; Berry and Bradley, 1976; Sotelo and Dusart, 2009). Our data support this, as PCs of control slice cultures did not reveal any significant changes in dendritic length or cell soma size after 48 h of incubation, indicating that PC growth apparently finished in this mature phase. For therapeutic targets, our study examined PCs of this age to gain new insight into the impact of VEGF after the maturation process. Here, no significant increase on PC dendritogenesis and somatogenesis was detected after VEGF administration. Thus, our data indicate that VEGF effects on PC dendritogenesis and somatogenesis are age-dependent and are restricted to immature PCs, whereas mature PC dendrites and somata are not susceptible to VEGF anymore. This downregulation of VEGF sensitivity is in line with different other recent studies such as glia cell development in CNS and growth cone development in PNS (Foehring et al., 2012; Wuestefeld et al., 2012).

Axitinb is a potent and selective inhibitor of VEGF receptor tyrosine kinases 1–3 (Hu-Lowe et al., 2008). A current study could show that axitinib has a high specificity to VEGFR-2 (Giddabasappa et al., 2016). Through immunohistochemistry, qPCR and *in situ* hybridization we decisively proved VEGFR-2 expression in PCs. By blocking through axitinib treatment, it is very likely that VEGFR-2 and its corresponding ligand VEGF mainly mediate crucial effects in developing PCs. During this time, the growth of PC dendrites was strongly reduced under axitinib incubation. The qPCR results show a clear reduction in VEGFR-2 mRNA, and are in line with the lack of effects on mature PCs. In the present study, we observed a significant reduction in dendritic length after axitinib administration, so endogenous VEGF apparently plays an important role for proper dendritogenesis in neonatal PCs. Only slight reductions of dendritogenesis parameters between control and axitinib treated slice cultures were perceived in the juvenile stage. Thus, our presented data indicate for the first time that in this phase of development, endogenous VEGF probably plays a more subordinate role in the physiological maturation progress of the dendritic arbor. We cannot exclude the impact of other members of the VEGF familiy in the maturation of PCs. Focusing on the most active biological form of the VEGF family, VEGF-A, the presented results verify the prominent significance of VEGF/VEGFR-2 interaction during this process. The data are in accordance with other inhibition studies, which investigated the impact of VEGF inhibitors on the survival and dendritic outgrowth of neonatal PCs using primary cultures of p1 mice cerebella (Cvetanovic et al., 2011). These experiments point out a significant reduction in the longest PC dendrite, so that low levels of VEGF negatively influenced survival and dendritogenesis in PCs. In summary, our present study demonstrates that low VEGF levels compromise, yet additional VEGF accelerates PC dendritogenesis and somatogenesis.

### Therapeutic Impact of VEGF

VEGF and their inhibiting antibodies and drugs already have a high relevance in clinical practice, as it is used as a therapeutical drug for tumor treatment (Fernando and Hurwitz, 2004; Hutson et al., 2013), or in diabetic retinopathy (Das et al., 2015), age-related macular degeneration (Solomon et al., 2014) and intracranial injuries (Talwar and Srivastava, 2014). Storkebaum et al. (2005) demonstrated that VEGF administration improves motor performance and halts motor neuron degeneration in ALS rats. Besides this, in the case of Parkinson’s disease, VEGF is applied for its neuroprotective function (Cui et al., 2011). Altered PC miRNA expression plays an important role in SCA1 pathogenesis. Because of an increase in miR-150 levels, the expression of VEGF in PCs is down regulated, which modulates disease pathogenesis (Rodriguez-Lebron et al., 2013). SCA1 is primarily characterized by degeneration of the spinocerebellar tracts and the loss of cerebellar PCs (Durr, 2010; Seidel et al., 2012). One recent study analyzed SCA1 and demonstrated the supportive function of VEGF during development (Cvetanovic et al., 2011). Recently, it was of great interest to discover the VEGF that acts as a stimulating growth factor in dendritogenesis and spinogenesis during development, as well as in adulthood. Profound knowledge of VEGF could allow therapeutic use of VEGF for a variety of human diseases that affect the neuronal dendrites such as Down syndrome, Rett syndrome and fragile X syndrome (Kaufmann and Moser, 2000). In addition, for patients with focal ischemic infarcts and their resulting neuronal destruction and degeneration, VEGF could be a promising agent. Our data strongly indicate that VEGF administration is able to promote dendrite maturation during development, but unfortunately, there is a loss of VEGF susceptibility on matured neurons.

## Author Contributions

All authors contributed equally in conception of the work, data acquisition and analysis, interpretation of data, drafting the work and final approval of the version to be published. Besides this they all agree to be accountable for all aspects of the work.

## Conflict of Interest Statement

The authors declare that the research was conducted in the absence of any commercial or financial relationships that could be construed as a potential conflict of interest.
