# Supplementary material for: Morphological Plasticity of Emerging Purkinje Cells in Response to Exogenous VEGF
Source: Front Mol Neurosci. 2017 Jan 30;10:2. doi: 10.3389/fnmol.2017.00002 (PMC5276996; doi:10.3389/fnmol.2017.00002)
Supplement: Supplementary file 1 [file Image_1.pdf]

Supplementary Material

Morphological plasticity of emerging Purkinje cells in response to exogenous VEGF

Leonard Herrfurth[1], Verena Theis [1], Veronika Matschke[1], Caroline May[2], Katrin Marcus[2], Carsten Theiss[1 CA]

[1]Institut für Anatomie, Abteilung für Cytologie, Ruhr-Universität Bochum, Bochum, Germany

[2]Abteilung für Medizinische Proteomik/Bioanalytik, Medizinisches Proteom-Center, Ruhr-University Bochum, Bochum, Germany

*** Correspondence:** Prof. Dr. Carsten Theiss; e-mail: carsten.theiss@rub.de

1 Supplementary Figures and Tables

1.1 Supplementary Figures

Supplementary Data

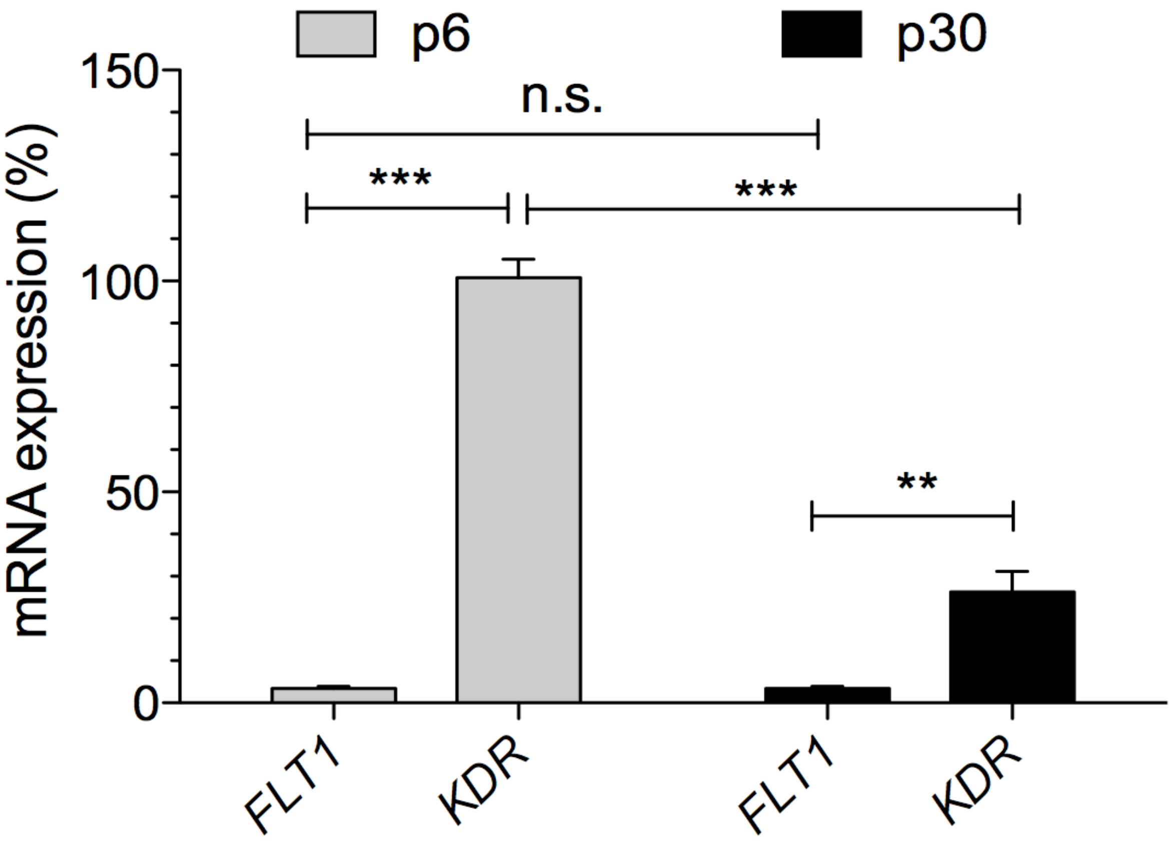

Figure 1: mRNA expression levels in PC

For relative quantification of *FLT1* and *KDR* expression in p6 and p30 rat cerebella, the $2^{-\Delta\Delta Ct}$ method was conducted using the housekeeping gene *GAPDH* for normalization; data are provided as means ± SEM. Data were tested for significance using Student's t-test. Significant differences are indicated by ***p<0.0001; n = 3; n.s. = not significant.
